# TRPS1 Is Differentially Expressed in a Variety of Malignant and Benign Cutaneous Sweat Gland Neoplasms

**DOI:** 10.3390/dermatopathology10010011

**Published:** 2023-02-02

**Authors:** Hatice B. Zengin, Chau M. Bui, Kristin Rybski, Tatsiana Pukhalskaya, Bahadir Yildiz, Bruce R. Smoller

**Affiliations:** 1 Department of Pathology and Laboratory Medicine, University of Rochester School of Medicine and Dentistry, Rochester, NY 14642, USA; 2 Department of Pathology and Laboratory Medicine, Cedars-Sinai Medical Center, Los Angeles, CA 90048, USA; 3 Department of Pathology and Laboratory Medicine, University of California, San Francisco, CA 94143, USA

**Keywords:** TRPS1, sweat gland tumors, eccrine gland

## Abstract

Neoplasms of sweat glands and the breast may be morphologically and immunophenotypically similar. A recent study showed that TRPS1 staining is a highly sensitive and specific marker for breast carcinoma. In this study, we analyzed TRPS1 expression in a spectrum of cutaneous sweat gland tumors. We stained five microcystic adnexal carcinomas (MACs), three eccrine adenocarcinomas, two syringoid eccrine carcinomas, four hidradenocarcinomas, six porocarcinomas, one eccrine carcinoma-NOS, 11 hidradenomas, nine poromas, seven cylindromas, three spiradenomas, and 10 syringomas with TRPS1 antibodies. All of the MACs and syringomas were negative. Every cylindroma and two of the three spiradenomas demonstrated intense staining in cells lining the ductular spaces, with negative to relatively weak expression in surrounding cells. Of the 16 remaining malignant entities, 13 were intermediate to high positive, one was low positive, and two were negative. From the 20 hidradenomas and poromas, intermediate to high positivity was revealed in 14 cases, low positivity in three cases, and negative staining in three cases. Our study demonstrates a very high (86%) expression of TRPS1 in malignant and benign adnexal tumors that are mainly composed of islands or nodules with polygonal cells, e.g., hidradenomas. On the other hand, tumors with small ducts or strands of cells, such as MACs, appear to be completely negative. This differential staining among types of sweat gland tumors may represent either differential cells of origin or divergent differentiation and has the potential to be used as a diagnostic tool in the future.

## 1. Introduction

Sweat glands and mammary glands share similar embryologic origins, as both derive from ectodermal appendages [1]. These two types of glands are considered to be structural and functional homologues of each other [1]. Therefore, neoplasms of sweat glands and the breast can show striking similarities in terms of morphologic as well as immunophenotypic presentation [1]. This resemblance can cause many challenges, especially in differentiating cutaneous metastases of breast cancer (CMBC) from primary sweat gland malignancies [2]. The treatment and prognoses of these tumors differ significantly, and this makes accurate diagnosis critical [2].

The *trichorhinophalangeal syndrome-1 (TRPS-1) gene*, also called the “*transcriptional repressor GATA binding 1*” gene, belongs to the GATA transcription factor family and functions as a transcriptional repressor [3]. It has been shown to be a ductal epithelial cell-specific gene expressed in normal breast glands and overexpressed in a significant portion of breast cancers [4]. A recent study demonstrated the immunohistochemistry (IHC) of TRPS1 in various breast tumors and proposed this IHC stain as a highly sensitive and specific marker for mammary tumors [5]. Our goal is to investigate TRPS1 expression in a variety of cutaneous sweat gland tumors (SGTs). In cases of negative staining, TRPS1 may be used to differentiate malignant SGTs from metastatic breast tumors. In cases of positive staining, we aimed to evaluate possible divergent staining patterns among SGTs.

## 2. Materials and Methods

This Institutional Review Board (IRB)-approved study was based on a retrospective analysis of archival tissue. Surgical pathology specimens diagnosed with “microcystic adnexal carcinoma”, “eccrine carcinoma”, “hidradenocarcinoma”, “porocarcinoma”, “hidradenoma”, “poroma”, “cylindroma”, “spiradenoma”, and “syringoma” between 1 January 2005 and 31 December 2018 were selected for review. All selected cases were diagnosed by the dermatopathologists in our institution. The cases were reviewed by the authors for inclusion, with 10 eccrine carcinomas identified and recategorized based on histologic findings of the authors. Of these cases, 1 was defined as eccrine carcinoma not otherwise specified (NOS). Overall, 5 cases of microcystic adnexal carcinoma (MAC), 3 cases of eccrine adenocarcinoma, 2 cases of syringoid eccrine carcinoma, 4 cases of hidradenocarcinoma, 6 cases of porocarcinoma, 1 case of eccrine carcinoma NOS, 11 cases of hidradenoma, 9 cases of poroma, 7 cases of cylindroma, 3 cases of spiradenoma, and 10 cases of syringoma were included in this study.

Immunohistochemical studies were performed on 4 µm sections of formalin-fixed, paraffin-embedded tissue using a Leica Bond III instrument. The IHC assay consisted of a rabbit monoclonal antibody against human TRPS1 (clone EPR16171 from Abcam). Antigen retrieval was performed with Bond Solution #2 (pH 9.0). TRPS1 antibody with dilution 1:6000 was incubated for 8 min at room temperature. Tissue staining was performed on a Leica BOND III immunostainer with a Leica Refine Polymer Detection Kit. A breast carcinoma HER2 control kit was used as an external positive control for TRPS1.

The positivity of TRPS1 was defined as dark-brown nuclear staining. The percentage of immunoreactive cells was graded as follows: 0, <1%; 1, 1–10%; 2, 11–50%; 3, 51–100%. Staining intensity was graded as follows: 0, negative; 1, weak; 2, moderate; 3, strong. Immunoreactivity scores were calculated by multiplying the number corresponding to the percentage of immunoreactive cells by the number corresponding to staining intensity. The immunoreactivity scores were reported as negative (0–1), low positive (2), intermediate positive (3–4), or high positive (6 and 9) for TRPS1 expression [5]. If more than one intensity group was present with different percentages, the highest score was given to those cases. Cylindroma and spiradenoma cases were not subjected to the reporting system due to their unique staining patterns.

## 3. Results

We evaluated TRPS1 IHC staining in a total of 61 skin biopsy specimens and report 51 of them based on the above criteria (Table 1).

**Figure 1:**
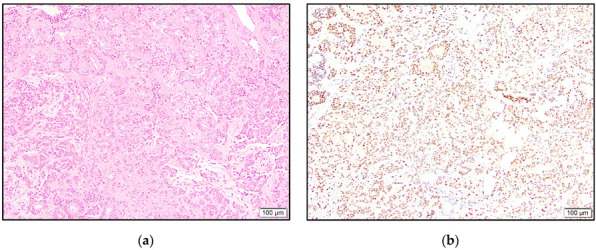
Eccrine adenocarcinoma with glandular architecture (**a**) and high positive TRPS1 expression (**b**).

In the malignant tumors, all three eccrine adenocarcinomas showed intermediate to high positivity (Figure 1). One of these cases revealed accentuated staining of the cells lining ductular spaces. The eccrine carcinoma NOS case was high positive.

**Figure 2:**
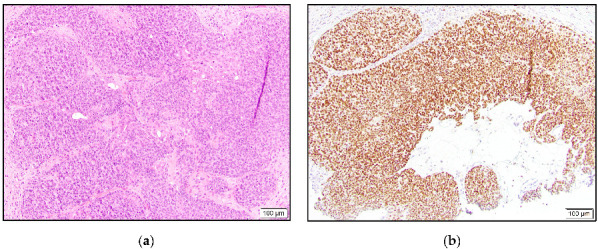
Hidradenocarcinoma with dermal tumor nodule composed of eosinophilic cells and mitoses (**a**) and high positive TRPS1 expression (**b**).

Of the two syringoid eccrine carcinomas, one was intermediate positive, and the other case was negative. Three of our hidradenocarcinomas were high positive (Figure 2), and one case was negative for TRPS1 staining.

**Figure 3:**
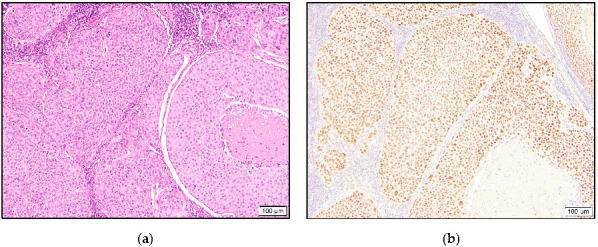
Porocarcinoma with back-to-back tumor nodules comprising occasional ducts and eosinophilic tumor cells (**a**) that stained positive for TRPS1 (**b**).

In the porocarcinoma group, all six cases demonstrated TRPS1 expression, with five being intermediate to high positive (Figure 3).

**Figure 4:**
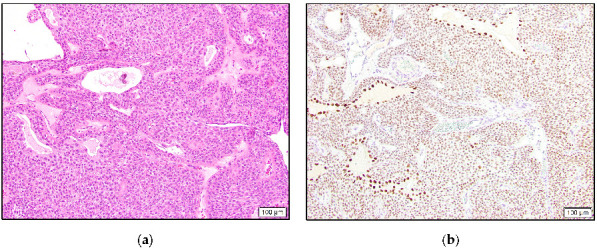
Hidradenoma with solid and cystic areas composed of eosinophilic cells and focal clear cells (**a**) and TRPS1 expression with accentuated staining in cells lining cystic spaces (**b**).

In the benign category, eight hidradenoma cases were intermediate to high positive, one was low positive, and two were negative. Similar to the eccrine adenocarcinoma case, four hidradenomas showed stronger staining in the cells lining cystic or ductular spaces (Figure 4).

**Figure 5:**
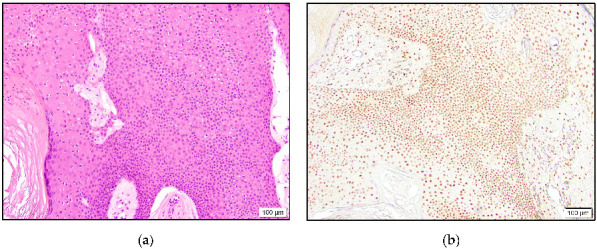
Poroma with proliferation of monotonous basophilic cells (**a**) and high positive TRPS1 expression (**b**).

In the poroma group, six and two cases demonstrated intermediate to high and low positivity, respectively (Figure 5). One poroma was negative for TRPS1 expression. In the majority of the positive cases, variable staining intensity was present in the tumor cells. This finding was more evident in the hidradenomas.

**Figure 6:**
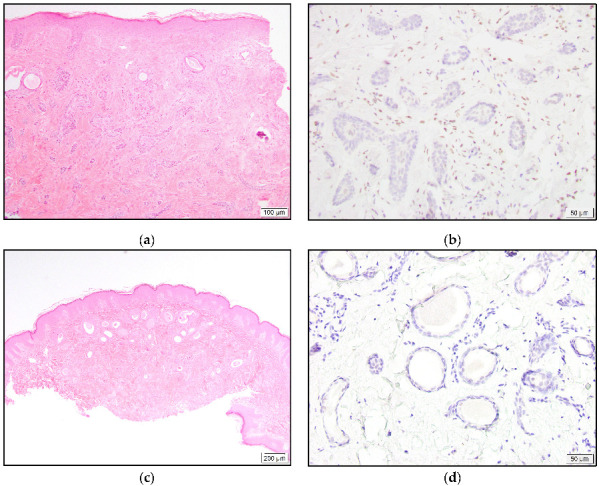
Infiltrative growth pattern of MAC with cords of small cuboidal cells and occasional duct formation (**a**). Negative TRPS1 staining in malignant cells and dermal mesenchymal cells, with positive staining (**b**). Well-circumscribed syringoma with tadpole-shaped ducts (**c**). Lack of TRPS1 expression in neoplastic cells (**d**).

All MAC and syringoma cases were negative for TRPS1 expression (Figure 6).

**Figure 7:**
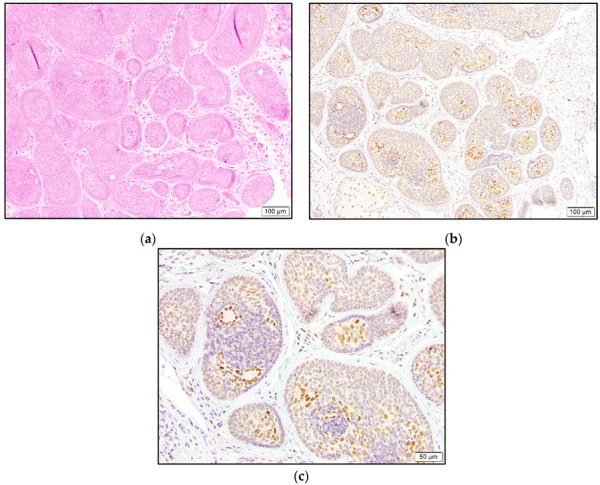
Cylindroma with islands of basaloid cells arranged in a jigsaw puzzle pattern (**a**). Strongest TRPS1 staining in luminal cells, weaker staining in surrounding neoplastic cells, and foci of negative staining with predilection for palisading cells (**b**,**c**).

The majority of the cylindroma and spiradenoma cases showed distinctive staining patterns and were not categorized by the abovementioned reporting system. Only one spiradenoma was completely negative. The remaining two spiradenomas and all seven cylindromas revealed the strongest staining in the luminal cells and relatively weaker or negative staining in the surrounding neoplastic cells. The outermost layer with palisading cells was mostly negative for TRPS1 expression (Figure 7).

**Figure 8:**
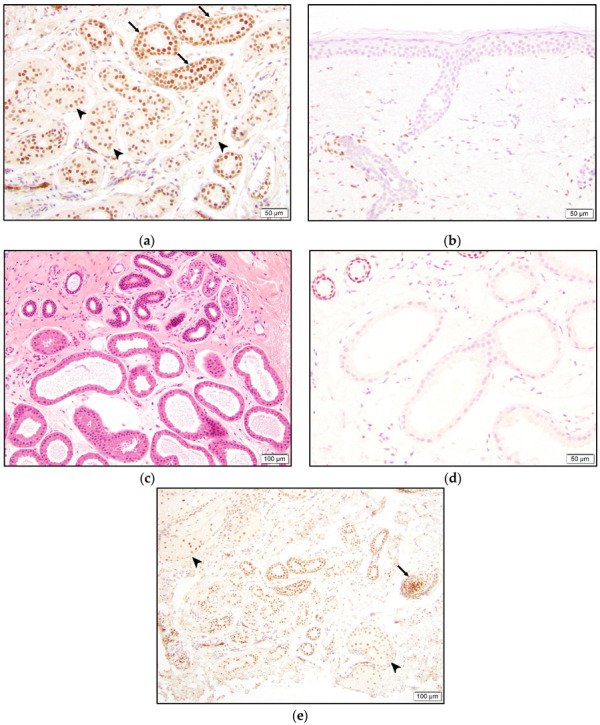
More intense TRPS1 expression in the two eccrine gland sweat duct cell layers (arrow) compared to the inner cell layer of the secretory coil (arrow-head) (**a**). Negative TRPS1 staining in acrosyringium (**b**). Benign apocrine and eccrine glands (**c**) with negative and positive TRPS1 expression, respectively (**d**). TRPS1 positivity in hair follicle bulb with papillary mesenchymal body (arrow) and sebaceous gland (arrow-head) (**e**).

TRPS1 staining was also observed in normal structures. The inner cell layer of the eccrine gland secretory coils and the two cell layers of the eccrine ducts were positive for TRPS1. Ductular cells seemed to stain darker than secretory cells (Figure 8). However, acrosyringium was negative for TRPS1 expression (Figure 8). Interestingly, the apocrine glands did not stain with TRPS1 (Figure 8). Strong staining was also present in hair follicles, especially in the bulb and papillary mesenchymal bodies (dermal papilla). Additionally, the majority of the squamous epithelium, sebaceous glands, and fibroblasts, mainly around hair follicles, demonstrated staining to varying intensities (Figure 8).

## 4. Discussion

Poroma and syringoma are categorized as benign eccrine SGTs [6]. Hidradenoma represents a “nosological jungle” and comprises both eccrine- and apocrine-originated tumors [7]. Cylindroma and spiradenoma are benign tumors with debatable eccrine gland or hair follicle origin [6,8]. Eccrine carcinomas (malignant SGTs) include syringoid eccrine carcinoma, eccrine adenocarcinoma, hidradenocarcinoma, porocarcinoma, and MAC, among others [6]. These entities exhibit distinct histologic features [7]. Excluding syringoma and MAC, the remaining tumors generally comprise single or multiple islands or nodules of round to polygonal cells with basophilic, eosinophilic, or clear cytoplasm [7]. Syringoma and MAC both show strands or cords of cells, often with duct formation described as “tadpole” in shape [7].

Malignant SGTs constitute the biggest pitfall in diagnosing CMBC [2]. TRPS1 IHC was reported to show reactivity with breast carcinoma [5]. Our study demonstrates a very high expression of TRPS1 in eccrine carcinomas other than MAC (88% overall; 81% intermediate to high positive). Although we used a different IHC clone than that in a previous study [5], our findings show that TRPS1 is possibly not helpful in differentiating malignant eccrine tumors from CMBC. Another malignant SGT, MAC, did not show TRPS1 expression in any cases. Nevertheless, MAC is less likely to be in the differential of CMBC.

Eccrine glands comprise secretory coils and sweat ducts, which are further divided into intraglandular, intradermal, and intraepidermal (acrosyringium) segments [9,10]. The secretory coil comprises two main cell types: inner secretory cells and outer myoepithelial cells [9]. CK7 is known to be a secretory cell-specific marker, while SMA (more sensitive) and CD10 selectively stain myoepithelial cells [11]. On the other hand, sweat ducts are lined by two cell layers and show a different IHC profile compared to coils with CK6 and CK10 expressions [11]. A comprehensive report on keratin expression in eccrine sweat glands also revealed divergent, complex keratin patterns in the various tissue units of the sweat gland [10]. Each unit of the eccrine sweat gland, including the segments of the sweat duct, expresses at least one keratin family member that serves as a tissue-specific marker [10]. One can conclude that differential expression of other antigens can be expected in each segment of the sweat duct and secretory coil. In this study, we observed TRPS1 positivity in secretory cells and (slightly stronger) in the two duct cell layers. However, acrosyringium appeared to be negative.

Eccrine tumors arise from secretory coils and/or ducts. There are multiple studies in the literature focusing on the derivation and differentiation of benign eccrine tumors with variable IHC stains [11,12,13,14]. In hidradenomas, both luminal cells and peripheral polygonal cells have shown concomitant secretory coil and inner ductular cell differentiation [12]. The differentiation has shown to be more conspicuous in luminal cells [12]. In this study, a total of nine hidradenoma cases, with eight intermediate to high positive, expressed TRPS1. We observed a more discernable spectrum of staining intensity among tumor cells in hidradenoma, which is possibly consistent with both secretory (weaker) and ductular (stronger) cell differentiation. In four cases, accentuated staining was prominent in either the entire or the majority of luminal cells, which may suggest relatively selective ductular differentiation in these areas. Myoepithelial cells are favored not to be involved in this entity [12,14]. Because myoepithelial cells are negative for expression, the TRPS1 stain does not contribute to this discussion.

A previous study on the differentiation of poromas showed ductular-type staining (CK6 and CK10) of luminal cells within the tumor but failed to identify the origin of peripheral cells [11]. A more comprehensive study proposed that poromas mainly arise from or differentiate towards the outer cells of eccrine ducts [12]. The authors also hypothesized inner ductular cell or secretory cell differentiation during lumen formation based on the IHC panel used [12]. Multiple studies have agreed on the absence of myoepithelial cells in poromas [11,12,13]. In this study, the percentage of TRPS1-positive cases was highest in poromas (~90% in total). However, we did not notice any specific staining pattern to be able to comment on particular differentiations. This finding may be in keeping with the relatively exclusive differentiation towards outer ductular cells.

Cylindromas and spiradenomas display similar histologic and immunohistochemical features [11,12]. Some authors consider these two entities morphological variants of the same tumor [15]. Although their origin is debatable, data that support eccrine lineage mostly indicate ductular differentiation in luminal cells and secretory coil differentiation in the surrounding neoplastic cells [11,12]. Multiple studies have shown myoepithelial marker expression at the periphery of tumors, especially in the outermost palisading cells [12,13,14]. Although Missall et al. failed to show myoepithelial differentiation in cylindromas and spiradenomas, they reported negative staining of peripheral and palisading cells with multiple secretory coil and duct markers [11]. In our study, luminal cells revealed stronger staining compared to the remaining tumor cells in all cylindromas and two spiradenomas. We also observed negative TRPS1 expression with peripheral arrangement in the majority of cases, which suggests possible myoepithelial or, at least, non-eccrine lineage in those cells. Considering the mild intensity difference between the secretory coil and ductal cells, our staining pattern also supports ductular differentiation in luminal cells and secretory coil differentiation in surrounding cells. On the other hand, another study showed hair-follicle stem cell marker expression in cylindromas and spiradenomas and postulated that these tumors originate from bulges in the hair follicle region [8]. Our study revealed TRPS1 positivity in hair follicles and potentially indicates follicular differentiation in these entities as well.

Syringomas and MACs show similar histologic findings and are commonly in each other’s differentials [7]. Syringomas are believed to derive from or differentiate towards eccrine ducts due to their staining pattern [11,12]. In contrast, MACs are known to express CK7 in luminal cells and SMA in peripheral cells, which suggests a secretory coil origin [16]. Although we demonstrate TRPS1 staining in secretory cells and in ductular cell layers, none of our syringomas or MACs were positive for the expression. Alternatively, these two tumors may arise from the acrosyringium, which also showed negative expression of TRPS1. A consistent lack of TRPS1 staining in these entities may suggest a loss of expression during the neoplastic process. However, syringomas and MACs are well-differentiated tumors, which calls this hypothesis into question. A different cell of origin, such as myoepithelial cells, may also explain our findings, especially for MACs. Yet this theory seems to be less likely in syringomas, as multiple studies have shown the absence of myoepithelial differentiation by various markers [11,12,13].

Mammary glands are often defined as “modified” apocrine glands. In light of normal TRPS1 staining in benign breast luminal cells [5] and eccrine ducts, its negative expression in apocrine glands was an unexpected finding. Because the *TRPS1* gene is known to be important for the growth and differentiation of normal mammary epithelial cells, this gene may be the actor behind the “modification”. Nonetheless, this differential staining will pave the way for a more detailed investigation of sweat gland tumor origins. It may also help in the differentiation of CMBC from apocrine sweat gland tumors.

Lastly, the TRPS1 gene is known to be important in hair follicle development, and its expression was previously shown in the dermal papilla and the mesenchymal cells surrounding the hair follicle in murinae [17]. Our study also shows TRPS1 expression in the dermal papilla and dermal mesenchymal cells, especially adjacent to hair follicles, and in the bulb of the hair follicle in humans.

## 5. Conclusions

TRPS1 expression is not specific to breast cancer, and a high percentage of SGTs (86% overall; 75% intermediate to high) also show positivity. It does not have the function of differentiating benign from malignant SGTs, as positive expression can be seen in both. In light of the natural TRPS1 expression in normal eccrine glands, we think that negative staining in SGTs other than syringoma and MAC may be due to differential fixation or tissue age. Unlike malignant SGTs, benign tumors exhibit distinctive staining patterns, possibly based on their origin or divergent differentiation. TRPS1 can be another sensitive IHC marker for certain SGTs and has the potential to be used as a diagnostic tool. However, our limited sample size necessitates further studies with larger case groups.

## Figures and Tables

**Table 1:** Number of cases in each diagnostic group and their distribution among result categories.

Entity	Total Number of Cases	Negative (n)	Low Positive (n)	Intermediate Positive (n)	High Positive (n)
MAC	5	5	0	0	0
Eccrine adenocarcinoma	3	0	0	2	1
Syringoid eccrine carcinoma	2	1	0	1	0
Eccrine carcinoma, NOS	1	0	0	0	1
Hidradenocarcinoma	4	1	0	0	3
Porocarcinoma	6	0	1	2	3
Hidradenoma	11	2	1	1	7
Poroma	9	1	2	3	3
Syringoma	10	10	0	0	0

MAC: Microcystic adnexal carcinoma; n: number; NOS: Not otherwise specified.

## Data Availability

The data presented in this study are available on request from the corresponding author.
